# Lessons from Animal Models in Sjögren’s Syndrome

**DOI:** 10.3390/ijms241612995

**Published:** 2023-08-20

**Authors:** Diana Mieliauskaitė, Vilius Kontenis, Almantas Šiaurys

**Affiliations:** 1 State Research Institute Center for Innovative Medicine, Department of Experimental, Preventive and Clinical Medicine, LT-08406 Vilnius, Lithuania; vilius.kontenis@imcentras.lt; 2 State Research Institute Center for Innovative Medicine, Department of Immunology, LT-08406 Vilnius, Lithuania; almantas.siaurys@imcentras.lt

**Keywords:** animal models, genetic mouse model, induced mouse model, humanized mouse model, Sjögren’s syndrome, microbiota, microbiome

## Abstract

Primary Sjögren’s syndrome (pSS) is a connective tissue disease characterized by a wide spectrum of clinical features, extending from a benign glandular disease to an aggressive systemic disorder and/or lymphoma. The pathogenesis of Sjögren’s syndrome (SS) is not completely understood, but it is assumed that pathogenesis of SS is multifactorial. The studies based on the animal models of SS provided significant insight in SS disease pathogenesis and management. The aim of this review is to summarize current studies on animal models with primary SS-like symptoms and discuss the impact of these studies on better understanding pathogenesis and management of Sjögren’s syndrome. Databases PubMed, Web of Science, Scopus and Cochrane library were searched for summarizing studies on animal models in SS. Available data demonstrate that animal models are highly important for our understanding of SS disease.

## 1. Introduction

Primary Sjögren’s syndrome (pSS) is a connective tissue disease characterized by a wide spectrum of clinical features, extending from a benign glandular disease to an aggressive systemic disorder and/or lymphoma [1,2,3,4]. According to a meta-analysis, the pooled incidence rate of pSS was estimated to be 6.92 (95% CI, 4.98–8.86) per 100,000 people/year, and the prevalence rate was 60.82 (95% CI, 43.69–77.94) cases per 100,000 inhabitants. Regarding the prevalence of SS in Europe, it was analyzed three robust epidemiological studies and estimated a combined prevalence of nearly 39/100,000 (0.04%) [5,6]. Primary Sjögren’s syndrome mainly affects women (average female to male ratio 9:1) of the middle age, with a mean age ranging from 51.6 (±13.8) to 62 (±13) years [7]. There is also evidence that the disease is initiated long before clinical symptoms are visible, since autoantibodies can be detected years in advance [8].

The pathogenesis of Sjögren’s syndrome is not completely understood, but it is assumed that pathogenesis of SS is multifactorial. Genetic features, including certain HLA phenotypes and polymorphisms in genes encoding cytokines or factors implicated in cytokine signaling, environmental (such as infections, chemical and physical agents, life-style), hormonal factors, as well as stressful life events, are thought to participate in autoimmune rheumatic diseases’ pathogenesis [9,10,11,12,13,14]. The studies based on the animal models of SS provided significant insight in SS disease pathogenesis and management. The aim of this review is to summarize current studies on animal models with primary SS-like symptoms and discuss the impact of these studies on better understanding pathogenesis and management of Sjögren’s syndrome. Databases PubMed, Web of Science, Scopus and Cochrane library were searched for summarizing studies on animal models in SS.

## 2. Animal Models in Sjögren’s Syndrome Pathogenesis Studies

The pathogenesis of SS is multifactorial, and SS disease is characterized by a wide spectrum of clinical features, extending from a benign glandular disease to an aggressive systemic disorder and/or lymphoma. It is recognized that exposure to certain environmental factors can play a crucial role in the immune dysregulation of susceptible individuals, leading to the onset of SS. Disruption of innate immune barriers plays a crucial role in the pathogenesis of SS, especially in the early stages of the disease. However, the adaptive immune system plays a key role in the development of SS. Continuous activation of B cells and proliferation of Th1 and Th17 cells contribute to disease progression. Recently, researchers have focused on epithelial cell function, which has been shown to be an important factor in the pathogenesis of this complex disease [12,15,16].

Genetic susceptibility plays an important role in the pathogenesis of SS. Previous studies have used SS genome-wide association analysis to identify IRF5-TNPO3, STAT4, IL12A, FAM167A-BLK, DDX6-CXCR5 and TNIP1 as risk sites and IRF5 and STAT4 as SS susceptibility genes [17]. GTF2I and RBMS3 were found to be the two most important susceptibility genes for pSS in women, which explains the high incidence of pSS in women. Long non-coding RNA (lncRNA) regulates gene expression but does not encode functional proteins, affecting chromosomes, DNA, transcription and post-transcriptional modifications. incRNA also regulates the differentiation and development of immune cells and the production of pro-inflammatory mediators, thereby affecting the development of autoimmunity. Many lncRNAs may be responsible for the increased risk of SS, as they are dysregulated in pSS patients. Research results have shown that miRNA-181a, miRNA-16, miRNA-146a and miRNA-155 play an essential role in the pathogenesis of pSS. Additionally, epigenetics such as DNA methylation and histone modification may be involved in the development of SS [18].

All existing animal models reveal different aspects of the disease, and all of this information needs to be supplemented and translated with care to give a more accurate picture of the pathogenesis of the disease. Depending on their role in the SS animal models, the factors contributing to the pathogenesis of the disease can be divided into two main categories: disturbed homeostasis in the exocrine glands and in the immune system.

### 2.1. Dysregulated Homeostasis in Exocrine Glands

Salivary gland epithelial cells (SGECs) in the SS are crucial for the initiation and development of the immune response. Abnormal homeostasis of exocrine glands is considered to be the initial step in the development of SS and exocrine dysfunction precedes inflammation in SS. After antigen stimulation, SGECs release anti-inflammatory factors that induce infiltration of immune cells and act as antigen-presenting cells to stimulate T lymphocyte differentiation [19,20]. CD80/86 is expressed on the surface of SGECs from SS patients. Treatment of an NFS/sld mouse model of SS with anti-CD86 antibodies ameliorates the autoimmune damage of salivary and lacrimal glands, suggesting that these epithelial cells may have antigen-presenting functions and may increase T cell proliferation and activation, leading to glandular damage [18]. TLRs, an important class of protein molecules, are involved in innate immunity. These receptors are expressed on immune cells and some epithelial cells. Binding of TLRs to the receptor induces the production of type 1 IFN and other cytokines. In addition, there is an increased expression of TLRs on the SGECs in SS. These results indicate the role of SGECs in the pathogenesis of PSs. TLR8ko mice show salivary adenitis, cytokines and anti-SSA and anti-SSB autoantibodies, which were not present in TLR7/8KO mice, suggesting that these changes depend on TLR7, further confirming the important role of TLR7 signaling in the local and systemic manifestations of SS [21].

In human SS, salivary and lacrimal glands exhibit characteristic apoptosis of glandular epithelial cells. This glandular epithelial cell apoptosis has been observed in mouse models of SS, such as NOD-derived strains, Ar KO mice, RbAp48-tg mice, NFS/sld mice, STAT3 KO mice and TSP-1 KO mice [22,23,24,25]. RbAp48-tg mice show autoreactive T cells and autoantibodies, which lead to histological lesions and exocrine gland dysfunction. These findings have demonstrated that the apoptosis of glandular epithelial cells can trigger autoimmune responses in SS. In mouse models characterized by epithelial cell apoptosis, autoantibodies against the proteolytic fragments of 120 kD α-fodrin are observed, suggesting that autoimmunity against α-fodrin is mediated by epithelial apoptosis. Therefore, mouse models demonstrated that the axis of epithelial apoptosis/α-fodrin proteolysis/autoimmunity against α-fodrin plays an important role in the development of SS [26,27,28].

An important histological sign of SS is ectopic expression of Class II major histocompatibility complex (MHC II) molecules on glandular epithelial cells. Studies on genetic models of SS *(*IQI/Jic mice, Rb Ap48-tg mice) showed that ectopic expression of MHC II molecules is mediated by genetic factors. IFN-γ produced by salivary gland epithelial cells in *RbAp48-tg* mice plays an essential role in this process because it can induce expression of interferon regulatory factor 1 (IRF-1) and class II major histocompatibility complex transactivator (CIITA), which are primary regulators of MHC II molecules [29]. Ectopic expression of MHC II molecules on epithelial cells has been demonstrated in induced mouse models (*M3R* immunization induced, Ro60_316-335 peptide-induced mouse model), as well. Results of the Ro60_316-335 peptide–induced model studies demonstrate that ectopic expression of MHC II molecules can also be caused by environmental factors and ectopic expression of MHC II molecules might be involved in the development of disease [30]. Due to ectopic expression of MHC II molecules, glandular epithelial cells might act as antigen-presenting cells and contribute to the development of SS-like disease.

Abnormal proliferation of glandular epithelial cells in addition to apoptosis and ectopic expression of MHC II molecules might also promote autoimmunity. Data of the studies with genetic mice models of SS indicate that the proliferation and functional perturbation of ductal epithelial cells might be the primary event mediating autoimmunity and lymphocyte infiltration in SS [30].

### 2.2. Dysregulated Homeostasis in the Immune System

*Dendritic cells (DCs)* initiate adaptive immune responses, including the generation of autoreactive T cells and regulatory T cells (Tregs). This role of DCs in the development of SS-like disease is supported by induced models by immunization by antigens (salivary gland proteins, the Carbonic anhydrase II (CAII) protein, M3R, Ro60) and genetic mouse models (*TSP-1 KO*). Studies with induced mouse models have evidenced that DCs are involved in processing and presenting antigens to T cells [31,32]. A role of DCs is suggested in genetic mice models due to enhancing the expression of co-stimulatory molecules on DCs, leading to autoimmunity [33]. The *NOD* mouse model supports the following hypothesis: early NOD mouse model demonstrates DCs accumulation in the submandibular gland, which stimulates the initial T cells to initiate autoimmunity, leading to the infiltration of T cells in the submandibular gland. DCs stimulate the activation of primary T cells, and thus, cause inflammation cytokines and stimulates T cell infiltration in the salivary glands, leading to sialadenitis and SS change [18].

*T cells.* Previously, B cell dysfunction is considered to play a key role in the initiation and development of pSS. However, B cell depletion did not show significant effects in pSS patients [34]. T cells play important role in Sjögren’s syndrome pathogenesis due to abnormal activation and expansion of autoreactive T cells. These data are supported by findings from animal models. T cells are the predominant cell type in the inflammatory cell infiltrates in exocrine glands in all mouse models. The transfer of T cells from diseased mice in animal models induced SS-like disease in recipient immunodeficient mice, and demonstrated that T cells are indispensable for disease development. Deficiencies in T cells or cytokines associated with T cells prevent mice developing from SS-like disease. Mice with T cells specific P13K deficiency develop SS-like disease spontaneously. Several mouse models have shown that autoreactive T cells against M3R and 120 kD α-fodrin are potentially pathogenic. In summary, T cells are sufficient to induce and mediate disease, and are indispensable for disease development [35,36,37,38]. A large number of T cells, as well as serum anti-SSB and anti-SSA autoantibodies, are present in the salivary glands of *SATB1cKO* mice. *RAG2-/-* mice that received *SATB1cKO* mouse T cells (mice with RAG2 gene deletion cannot produce mature lymphocytes) had reduced salivary secretion and damaged salivary acinar cells, which suggest that T cells play an important role in the pathogenesis of SS [18].

Especially T helper 17 (Th17) cells and regulatory T (Treg) cells play a critical role in regulating the immune balance of systemic inflammation. Th17 cells are involved in a variety of autoimmune diseases, including SS, and the nuclear receptor retinoic acid-related orphan receptor (RORγt) is an essential transcription factor for the differentiation of Th17 cells. Salivary glands of RORγt transgenic (RORγt Tg*)* mice were stained with hematoxylin and eosin, which revealed sialadenitis due to excessive CD4+ T cell infiltration. The infiltration of CD4+ T cells and cytokines in the salivary gland was significantly increased compared to the control group. These results provide strong evidence for the involvement of RORγt and CD4+ T cell subsets in the pathogenesis of SS. Some studies used salivary gland protein to immunize wild-type mice and IL-17KO mice to establish SS mouse models. Wild-type mice immunized with salivary gland protein demonstrate SS symptoms; Th17 cells in the salivary glands increased. IL-17KO mice immunized with salivary gland protein did not exhibit SS symptoms or histopathological changes. Th17 cells produced in vitro were adoptively transferred to IL-17KO mice, which resulted in reduced salivary secretion and increased levels of serum autoantibodies [18].

Treg cells negatively regulate the immune processes. Studies have shown that although the exocrine glands of SS mouse models are infiltrated with Treg cells, SS-like symptoms still occur, which suggests that the number of Treg cells may not be sufficient to inhibit effector T cell-induced thymectomy in mice. A recent study observed a large number of CD8+ T cells in the exocrine glands of SS animal models and patients; removal of these resident CD8+ T cells after disease onset can alleviate the pathology.

Numerous murine models have demonstrated the implication of Th17/Treg cells imbalance in the disease induction. Th17/Treg cell imbalance can be attributed to the increased IL-6 level in the inflammatory environment. IL-6 synergizes with TGF-β to facilitate Th17 differentiation, while TGF-b in the absence of IL-6 promotes Treg differentiation. Treg cells in the lacrimal gland of C57BL/6.NOD.Aec1Aec2 mice are decreased, but Th17 cells and IL-17A expression are increased in comparison to those in the wild-type control mice in the early stage of pre-clinical disease [34].

Consistently, transient depletion of Treg cells results in enhanced salivary gland infiltration in *NOD* mice [34]. The effect of Th17/Treg cell imbalance on disease induction is further elucidated in thrombospondin-1 (TSP1)-depleted mice, an in vivo activator of latent TGF- β. These mice spontaneously present with ocular inflammation and dry eye symptoms, accompanied by elevated anti-SSA and anti-SSB antibodies. An increase in the number of splenic Th17 cells and an elevation in the levels of lacrimal IL-17 protein tend to coincide with a decrease in the number of splenic Treg cells in these mice. In vivo administration of TSP1 peptide can induce the formation of FoxP3þ Treg cells and reduce the number of Th17 cells in TSP1- knockout mice, thus alleviating disease symptoms. In NOD model mice with an altered MHC region (NOD.B10.H2b mice*)*, mice spontaneously develop ocular surface disease during aging. FoxP3þ Treg cells abnormally co-express transcription factors, such as Tibet and RORgt, to promote the production of IFN-g and IL-17 in these aged mice. Moreover, Treg cells from aged NOD.B10.H2b mice possess lower suppressive capacity than Treg cells from young mice. Transferring CD4þCD25þ Treg cells from these aged mice to T and B cell-deficient (RAG1-deficient) animals can induce the phenotype of periductal inflammation in the lacrimal glands, which is similar to the effect of transferring CD4þCD25 T helper cells [39,40]. These findings of murine models illustrate that Treg cells can acquire pro-inflammatory properties related to Th1 and Th17 cells, indicating that it is not only the enhanced pro-inflammatory properties of Th17 cells that promote disease [34].

*B cells.* Research studies from animal models revealed an important role of B cells in the development of SS-like disease. Genetic modifications directly affect B cells function and B cell dysregulation could lead to SS-like disease. Overactivation of B cells in the exocrine glands of SS patients manifests as gland swelling and the production of anti-SSA and anti-SSB autoantibodies [21]. C3H/HeJ mice immunized with Ro60-316-335 peptide spontaneously develop lacrimal gland inflammation and a variety of serum autoantibodies [18]. Additionally, B cell deficiency mediated by genetic modification or by anti-CD20 antibodies inhibits disease development in multiple models and this fact demonstrates an indispensable role of B cells. Notably, B cells participate in the impairment of secretory function via autoantibody production [32,41,42]. The C57BL/6.NOD-Aec1Aec2 mouse model of SS demonstrates increased specific chemokines, upregulation of chemokine receptors in the salivary glands, and infiltration of perivascular B lymphocytes, suggesting that B lymphocytes are crucial for the development of SS [43]. The chronic antigenic stimulation of B cells in SS, driven by known and yet unknown mechanisms, may lead to malignant transformation and generation of non-Hodgkin lymphomas (NHLs). The most frequent type of lymphoma in these patients is the extra-nodal marginal zone non-Hodgkin lymphoma MALT type (mucosa associated lymphoid tissue). Less frequently, higher-grade diffuse B-cell lymphomas and T-cell lymphomas have also been described [44]. Two mouse models (BAFF Tg, IL-14aTg) displayed a propensity for lymphoma development only, which however showed some similarities to the presentation of such lymphomas in SS patients. The TNF-/-BAFF-tg mice show a high prevalence (>35%) of B-cell lymphomas mainly in their cervical, inguinal or mesenteric lymph nodes, and also in their small intestine MALT type lymphoma. IL-14a transgenic mice overexpress IL-14a mainly in the B-cell compartment. The majority of these mice develop large B-cell lymphomas in their liver, gastrointestinal tract or lung, in a pattern that very much resembles the one seen in human SS-associated lymphomas [45,46,47].

*Cytokines* are synthesized and secreted by non-immune cells (endothelial cells, epidermal cells and fibroblasts) and immune cells (monocytes, macrophages, T cells, B cells and natural killer cells). Present studies have demonstrated that the combined action of various cytokines leads to the development of SS and promotes inflammation [48].

*Interleukins* are a class of cytokines produced and used by a variety of cells. IL-1 cytokines are key members of the IL-1 family. Studies on autoimmune regulation (*Aire*)-deficient mice have shown that IL-1 receptor knockout reduces ocular surface keratosis but not lymphocyte infiltration in mammary glands; therefore, IL-1 receptor therapy may only improve ocular symptoms [18].

IL-2 and IL-21 belong to the IL-2 family. Several studies have demonstrated that treatment with low-dose IL-2 increases the number of Treg cells compared to the baseline, decreases the Th17/Treg ratio and improves the symptoms of SS, indicating that IL-2 promotes the proliferation of Treg cells and suggesting that more attention should be paid to immune regulation for the treatment of SS [49]. Act1 attenuates IL-21-induced activation of STAT3, thereby inhibiting B cell activity. Mice lacking ACT1 exhibit SS-like symptoms [50]. It also affects IFN-1 signaling and plays an important role in the pathogenesis of SS [18].

The IL-17 family includes IL-17 and IL-25 (IL-17E). It has been reported that IL-17 produced by Th17 contributes to the development of SS [18]. However, it was later found that IL-17-deficient RORγt-Tg mice also spontaneously develop salivitis, which suggests that IL-17 is not necessary for the development of salivitis in RORγtTg mice [51].

IL-14 is a cytokine also known as B cell growth factor. Studies of transgenic mice with IL-14α suggest that IL-14 may play a role in the development of autoimmunity by regulating B cell function. IL-14a transgenic mice overexpress IL-14a mainly in the B-cell compartment, and they exhibit features of sialadenitis, hypergammaglobulinaemia, SLE-like nephritis and, rarely, positive autoantibodies (anti-SSA, anti-SSB, anti-dsDNA, anti-RNP, etc.), making them a possible model for SS secondary to SLE. These mice do not show any clinical relevance to human SS phenotype and female sex predisposition [45].

*The tumor necrosis factor (TNF) family* is a group of cytokines that cause cell death (apoptosis). TNF-α is an important contributor to SS pathogenesis and is secreted by CD4+ T cells, monocytes and epithelial cells. Overexpression of TNF-α induces symptoms of exocrine adenitis in mouse model, without serum autoantibodies [52].

*B cell-activating factor (BAFF)*, a member of the TNF superfamily, regulates immune responses. BAFF is a cytokine with important effects on the development and selection of B cells. A proliferation-inducing ligand (APRIL) is homologous to BAFF and is abnormally increased in the serum and inflammatory labial gland tissue of pSS patients. This fact indicates that APRIL participates in the pathogenesis of pSS by stimulating the proliferation of B cells. Additionally, it has been identified a highly conserved IFN-stimulated response element (ISRE) site near the BAFF gene promoter, which was functionally verified. IRFs combined with this site could regulate BAFF expression, production of B cells and production of a variety of autoantibodies, leading to the development of autoimmunity, such as PSs [18,53]. Additionally, varying degrees of acinar destruction and a high degree of lymphocyte infiltration were observed in the saliva and submandibular glands of BAFF Tg mice. BAFF transgenic C57BL/6 mice (BAFF-tg) overexpress B-cell activating factor, which results in B-cell hyperproliferation and systemic autoimmunity with lymphocytic infiltration in their salivary and lacrimal glands, decreased saliva production, SLE-like nephritis and increased production of rheumatoid factor and anti-DNA autoantibodies. These mice could serve as a mouse model of SS secondary to SLE. Finally, BAFF-tg mice exhibit neuroinflammation and anxiety-like behavior, which could probably mimic some of the neuropsychiatric symptoms seen in human SLE-SS. However, these mice do not produce anti-SSA, anti-SSB or other human-SS-specific autoantibodies, and do not develop dry eyes or symptoms from other organs/tissues (except from kidneys). They do not show a sex predisposition and they exhibit dry mouth only at older age [45].

*Interferon (IFN)* is a protein produced from different cell types and has powerful immunomodulatory effects. IFN-α and IFN-β are the most widely studied type I IFNs in SS [18,54]. IFN binds to its receptor and activates the JAK/STAT pathway. JAK inhibitors can treat pSS by downregulating the STAT pathway [55,56]. JAK inhibitors regulate pSTAT-1Y701 and pSTAT-3Y705 and activate STAT-3 proteins induced by IFN-α and IFN-γ in human SGECs. Studies have found increased type I IFN levels and type I IFN-induced gene overexpression in the salivary glands and peripheral blood of pSS patients, suggesting that the type I IFN pathway plays an important role in the pathogenesis of pSS [18]. Therefore, JAK inhibitors (such as tofatinib and baritinib) that act downstream of IFN may be used to treat SS. The results of a prospective, randomized, double-blind and multicenter clinical trial demonstrated that tofacitinib improves dry eyes in SS patients. Baritinib inhibits JAK/STAT signal transduction, IFN-γ-induced CXCL10 expression and immune cell chemotactic activity, indicating its effectiveness for the treatment of SS [55,56,57]. NOD.IFN-γ-/-, NOD.IFN-γ R-/- mice have been used to study the role of IFN-γ, in SS pathogenesis. Interestingly, NOD and NOD-derived mice develop disease symptoms spontaneously as a polygenic trait with a female predisposition, which resembles the development of SS in humans. However, none of them shows ectopic expression of MHC II, and apart from the NOD.B10.Hb mice, they do not show inflammatory infiltration in other organs (with the exception of pancreas in the NOD mice). Female C57BL/6.NOD-Aec1Aec2 *mice* do not show dry eyes, while the disease onset is delayed in comparison to their male counterparts [58]. Circulating IFN-α was significantly reduced in SS patients compared to healthy subjects. Several clinical studies have shown a significant increase in salivary production in SS patients treated with human IFN-α for 24 weeks. The mechanism of IFN-α production may be due to the upregulation of aquaporin 5 [18].

*Osteopontin (OPN)* is a multifunctional cytokine with diverse sources of production. An SS-like phenotype appears in OPN transgenic mice, which suggests that OPN is related to the etiology of SS [59]. Mice overexpressing osteopontin in bone tissue demonstrate reduced saliva production, lymphocytic infiltrations in almost half of their salivary and lacrimal glands, anti-SSA autoantibodies in their sera and increased levels of IL-4, IL-6, IL-2 and TNF-α, with female predisposition. Opn levels have been found to be increased both in salivary glands and sera of patients with SS. However, these mice do not show dry eyes, systemic symptoms or infiltration of other tissue [59,60].

*Neuroendocrine factors.* The incidence of SS is significantly higher in women than in men. In women, pSS onset usually occurs after menopause, probably because of insufficient estrogen secretion. In animal models, sex hormones affect humoral immunity and cellular immune response. Knockout of the mouse aromatase gene *(*ArKO mice) results in a lack of estrogen. These mice spontaneously develop an SS-like phenotype, characterized by B cell infiltration in the salivary glands, destruction of acinar cells and secretion of a variety of autoantibodies [18]. Aromatase deficiency causes M1 macrophages to produce proinflammatory cytokines in the adipose tissue and target organs, leading to SS-like lesions [61,62]. These findings suggest that estrogen plays a key role in the pathogenesis of SS. Studies demonstrated that TLR8ko female mice had more severe salivary adeninflammation compared to male mice. TLR7 expression was almost twice as high in the salivary glands of TLR8ko female mice compared to male mice. TLR7 gene is located on the X chromosome, which may partly explain the sex differences in SS pathogenesis [63]. B cells play a key role in the pathogenesis of SS. Leptin, a type of adipokine, induces B cells to secrete a variety of proinflammatory cytokines [64]. It is controversial whether leptin plays a role in the development of SS. The distribution of leptin and its receptors is not altered in the salivary glands of SS patients. Lymphocytic infiltration of the salivary glands is not related to leptin or its receptors, indicating that they are not significant in the pathogenesis of pSS [65]. However, some studies of mouse models reported that the levels of serum leptin and its receptors in the salivary glands are related to the severity of SS [66]. Therefore, leptin may be related to the development of SS. The CD25 null mouse model is an animal model of SS that involves the eyes and lacrist glands. Studies have revealed that, compared with wild-type mice, the CD25 null *SS* mouse model demonstrated fewer nerves in the cornea and reduced corneal sensitivity, leading to ocular symptoms of SS [67]. This suggests that changes in the nervous system are also involved in the presentation of SS symptoms.

The pathogenesis of SS is not well understood and treatment strategies are limited. SS animal models and patients share some common features of the disease and this contributes to the understanding of the pathogenesis of human SS. Consequently, no ideal animal model exists for SS. Mice models, particularly, are a powerful tool to elucidate the development of human SS [68].

Synteny between humans and mice is known to be significant, because less than 1% of mouse genes lack any homology to their human counterparts. Additionally, their small size, the easiness of their breeding and genetic manipulation, practicality, short lifespan, high fertility and a relatively low maintenance cost compared to bigger animal models have made mice perfect candidates for modelling human diseases [69]. For the studies of SS, according to the strategy of disease induction, animal models are divided into three categories: genetic models, in which animals develop disease’s signs spontaneously due to genetic mutations or modifications, induced models, in which disease is artificially induced in animals, and humanized models of SS, which has been developed with the injection of peripheral blood mononuclear cells from SS patients into immunodeficient NOD-scid IL2rγ mice [70,71,72]. Each different model mimics a specific aspect of the human SS disease (Table 1).

The creation of an ideal animal SS model will allow the determination of SS etiology and discovery of potential therapeutic targets and methods for the prevention of the disease’s complications. Such advances will improve the prognosis and quality of life of SS patients.

## 3. Insights on Microbiota in Sjögren’s Syndrome from Animal Models

The role of microbiota in human health and diseases is being highlighted by many studies since the discovery of microbiota, and research focusing on microbiomes has become a topic of great scientific and public interest. The current knowledge on the relationship between the human microbiome and SS reveals possible pathogenic mechanisms and forms a basis for future treatment options through influencing the microbiome.

Humans have coevolved with and harbor trillions of microbes from the time they are born. This collection of symbiotic, commensal and pathogenic microorganisms found on the skin, mucosal surfaces and other organs is referred to as the human microbiome. Microbiota can be classified into gut, oral, respiratory and skin microbiota. The microbial communities are in symbiosis with the host, contributing to homeostasis and regulating immune function. It is expected that people genetically predisposed to have autoimmune rheumatic disease can harbor gut microbiota capable of influencing the onset and severity of their disease. Evidence-based studies support that the microbiota can influence autoimmunity by molecular mimicry, epitope spreading or bystander activation [73,74,75,76,77]. Microbiota plays a notable role in the pathogenesis of Sjögren’s syndrome. Although until recently the causality between them was unknown, a new published study has provided evidence for causal effects of the composition of the gut microbiota and its related genes on SS risk: the presence of family *Porphyromonadaceae*, genus *Subdoligranulum*, genus *Butyricicoccus* and genus *Lachnospiraceae* is associated with a lower probability of developing SS, while genus *Fusicatenibacter* and genus *Ruminiclostridium9,* on the contrary, may increase the probability of SS [78].

The gastrointestinal microbiota can enhance the killing of pathogens by neutrophils and regulate the proliferation and differentiation of lymphocytes through their metabolites. It is revealed that segmented filamentous bacteria induce Th17 cells to secrete the pro-inflammatory cytokine interleukin-17 (IL-17) through serum amyloid A (SAA) produced by epithelia or signal transducer and activator of transcription 3 (STAT3) in epithelia, while polysaccharide A from *Bacteroides fragilis* activates Toll-like receptor 2 (TLR2) on fork head box P3 (FOXP3)-positive Treg cells to induce them to secrete anti-inflammatory cytokine IL-10 to antagonize the immune effect of IL-17 or other pro-inflammatory cytokines, which helps maintaining the balance of the immune system. Dysbiosis of gastrointestinal microbiota may break the balance and lead to primary Sjögren’s syndrome (pSS), which may be connected with chronic local gut mucosal inflammation, molecular mimicry and other mechanisms [79,80].

Animal studies have provided possible mechanisms for the association between SS and gastrointestinal microbiota [81]. Compared with normal mice, germ-free mice tended to develop dry eye and were accompanied by pSS-like symptoms, such as reduced density of conjunctival goblet cells, lacrimal lymphocyte infiltration and increased circulation levels of autoreactive T lymphocytes [82]. Possible mechanisms based on animal models that explain how gastrointestinal microbiome dysbiosis contributes to SS are molecular mimicry and chronic local gut mucosal inflammation.

**Figure 1:**
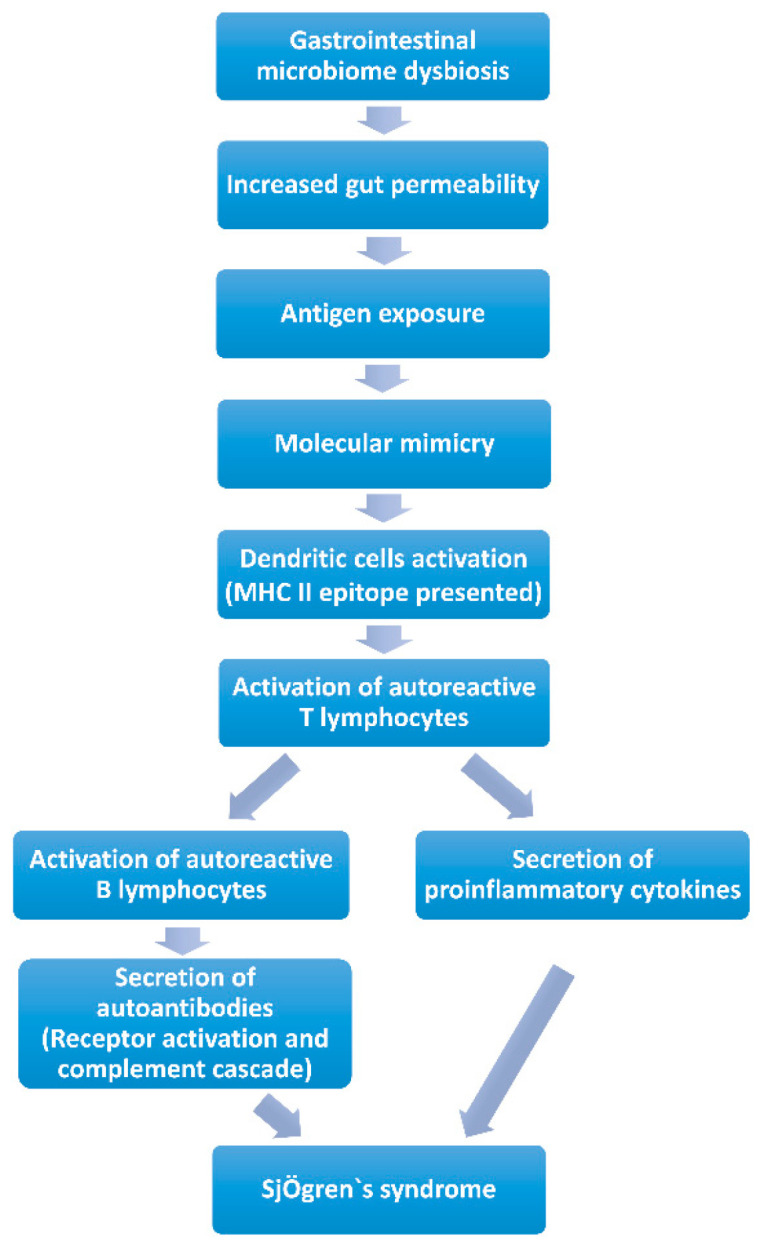
Contribution of gastrointestinal microbiome to Sjögren’s syndrome by molecular mimicry.

*Molecular Mimicry* (Figure 1). Molecular mimicry has been widely acknowledged as a mechanism of autoimmune diseases caused by infectious agents with epitope intermolecular spreading. B cell cross-reactivity between *Coxsackie virus* 2B protein and Ro60 has been reported to have significant association with the initiation and perpetuation of anti-Ro60 autoantibody response, which is essential in the pathology of SS. It is found that the serum of anti-Ro60-positive patients can react with the lysates of *Bacteroides thetaiotaomicron*. Additionally, a new antigen was discovered, named OmpA from *E. coli*, which elicits antibodies against SSA/Ro and SSB/La and causes inflammation of the salivary glands. These data suggest that microbe-originated molecules elicit inflammation in exocrine glands with specificity. Additionally, a peptide has been identified originating from *C. ochracea*, named von Willebrand factor type A domain protein (vWFA), which is the most potent activator of SSA/Ro60-reactive T cells, inducing robust IL-2 production. Recombinant vWFA protein and whole *E. coli* expressing vWFA are capable of initiating the activation of Ro60- reactive T cells. Besides, the highly-conserved microbial proteins of *Staphylococcus aureus* and *E. coli* can induce patients with SS to develop corresponding antibodies, and as a consequence of molecular mimicry, these antibodies cross-react against mitochondrial self-antigens [83]. Long-term gastrointestinal microbiome dysbiosis may result in increased intestinal permeability [84], which may increase the risk of gastrointestinal microbiome antigen exposure and autoantibody production [85]. The exposed gastrointestinal microbiome antigens are recognized and presented as MHC class II antigens by dendritic cells, which activate specific CD4 T lymphocytes [85]. The main mechanism involved is “molecular mimicry” [9]. Lysates, proteins or peptides that originate from bacteria and some other highly conserved proteins could react with anti-Ro60 antibodies or SSA/Ro60-reactive T cells, which are involved in the activation of excessive autoimmune response. Human Ro60 reactive T lymphocytes can react with Ro antigens and cross-react with von Willebrand Factor A (vWFA) and peptides containing the Trove domain [85]. These mimicking peptides are found in the human gastrointestinal microbiome (*Bacterioides finegoldii*, *Bacterioides intestinalis*, *Bacterioides fragilis* and *Alistes finegoldii*). When these microorganisms/or their antigens are exposed, Ro60-reactive T lymphocytes may be activated through “molecular mimicry” [81]. The activated specific autoreactive T lymphocytes reach the exocrine glands, such as the salivary and lacrimal glands, through the blood and secrete inflammatory cytokines (IFN-γ, IL-12, IL-17 and other), and contribute to salivary and lacrimal gland injury, inducing dry mouth and eye [9,85]. Activated specific autoreactive T lymphocytes promote the proliferation of plasma cells, which secrete autoantibodies such as SSA/Ro or SSB/La [77]. These autoantibodies recruit inflammatory cells to infiltrate exocrine glands and induce dry mouth and eye through receptor activation and the complement cascade [86]. Several studies have immunized mice with Ro polypeptides, and pSS-like symptoms were observed in these mice [87]. In addition, CD4 T lymphocytes, various pro-inflammatory cytokines and autoantibodies and abnormally high circulating levels of CD4+ T lymphocytes and B lymphocytes were found in the lacrimal glands of several mouse models of autoimmune dry eye [85,87,88].

**Figure 2:**
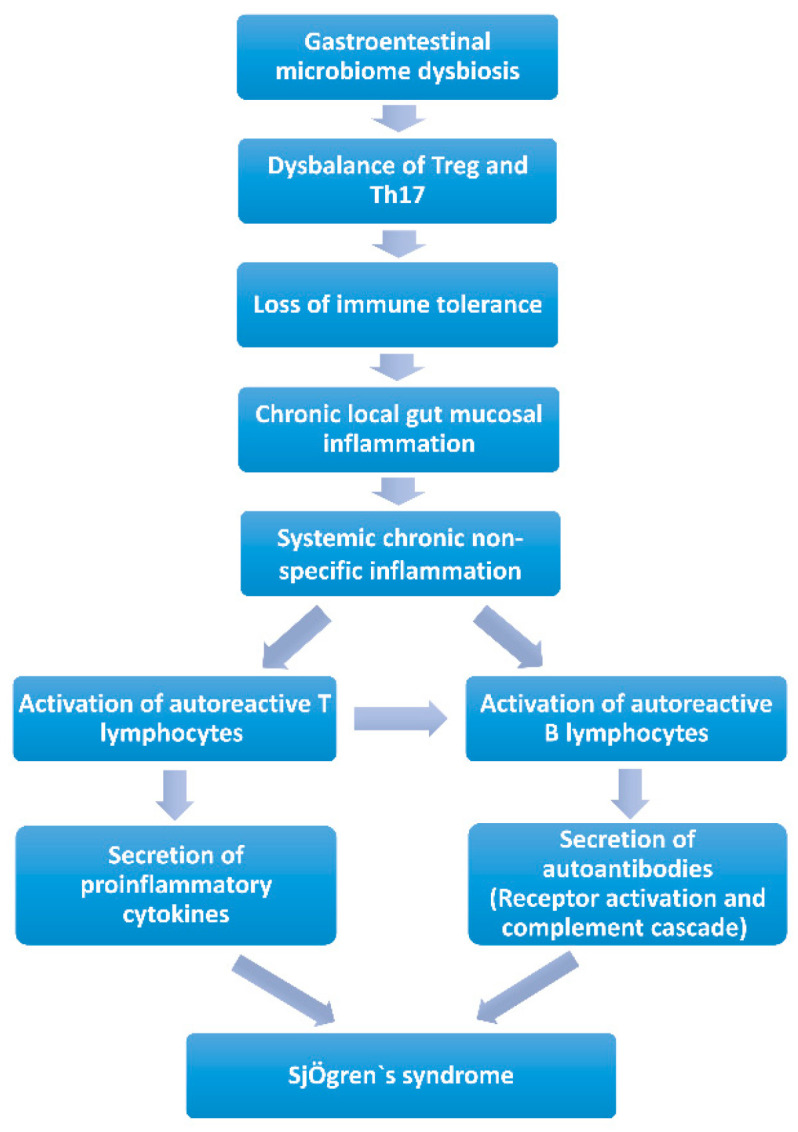
Contribution of the gastrointestinal microbiome to Sjögren’s syndrome by chronic local gut mucosal inflammation.

*Chronic local gut mucosal inflammation* (Figure 2). Long-term gastrointestinal microbiome dysbiosis causes the local intestinal mucosa inflammation. Lymphocytes and proinflammatory cytokines are released into circulation and may lead to systemic chronic non-specific inflammation, giving the potential to activate autoreactive T or B lymphocytes to induce autoimmunity [83,89,90]. Animal studies revealed that mice would develop chronic non-specific inflammation of the exocrine glands when exposed to chronic stimulation with *Escherichia coli* outer membrane protein A (OmpA) or flagellin (FliC), a flagellar filament structural protein. Additionally, pSS-like symptoms were observed [84,85]. Since many kinds of microorganisms in the gastrointestinal microbiota and their metabolites could maintain the state of immune tolerance by balancing the circulating fluxes of Treg and Th17, gastrointestinal microbiota dysbiosis would result in the loss of intestinal immune tolerance [91,92]. Short-chain fatty acids (SCFAs) produced by bacteria were decreased, which led to a reduction in the immune checkpoint molecules and an imbalance of T cells (Treg/Th17) and B cells (IL-10/IL-17 producing B cells), which provides a proinflammation microenvironment in SS, and induces chronic local gut mucosal inflammation, which causes systemic chronic non-specific inflammation [84,85,93].

Gastrointestinal microbiota dysbiosis and the imbalance between Treg/IL-10 and Th17/IL-17 were observed in the cornea, lymph nodes and serum of several mouse models of autoimmune dry eye [82,83,94,95]. Increased serum levels of IL-17, IL-6, IL-12, and TNF-α, decreased IL-10 serum levels and reduced expression of FOXP3 mRNA in peripheral blood monocytes were also observed in patients with pSS [96]. However, systemic chronic non-specific inflammation may require environmental factors such as stress and viral infection to trigger the critical activation of autoreactive T or B lymphocytes, followed by anti-SSA/Ro or anti-SSB/La antibody secretion and lacrimal gland injury [91,94,95]. If patients with chronic gastrointestinal microbiota dysbiosis are infected with the Coxsackie virus, systemic chronic non-specific inflammation in the body may enable the virus to amplify and maintain the autoimmune response through IFN-α and cause the occurrence of pSS [84,85]. These elements of evidence suggest the existence of a “gastrointestinal microbiota–lacrimal gland–ocular surface” axis, but the signal pathways or possible molecular mechanisms involved in each link of the axis still need to be confirmed and elucidated. Animal studies have provided evidence that gastrointestinal microbiome plays a causal role in the pathogenesis of pSS and have demonstrated the effectiveness of therapies such as probiotics and fecal bacteria transplantation for dry eye associated with autoimmunity [96].

However, large-scale human clinical studies and mechanistic studies are needed in the future to prove the causal relationship of the microbiome in SS.

Finally, available data demonstrate that animal models are highly important for our understanding of SS disease. All existing models shed light in different aspects of the disease and all these data must be complemented and translated cautiously to get a more spherical view of the SS disease pathogenesis.

## Figures and Tables

**Table 1:** Characteristics of mouse models used in the studies of Sjögren’s syndrome.

Mouse Model		Local Disease		Inflammatory Infiltration		Systemic Disease	Autoantibodies	
		Dry Mouth	Dry Eyes	SG	LG		Anti-SSA/Ro	Anti-SSB/La
Genetic mouse models	NZB/NZW F1	-	+	+	+	+	-	-
	MRL/lpr (MRL/Mp-lpr)	+	+	+	+	+	+	+
	NOD NFS/sld	+	+	+	+	+	+	+
	McH-lpr/lpr-RA1	+	-	+	+	+	-	-
	IQI/Jic	-	-	+	+	+	-	-
	Aly/aly	-	-	+	+	+	-	-
	Ar-KO	-	-	+	+	+	+	+
	Id3-KO	+	+	+	+	+/-	+	+
	T-cell specific PI3K-KO	-	+	-	+	+	+	+
	Act1-KO	+	+	+	+	+	+	+
	Aire-KO	-	+	+	+	-	-	-
	Erdj5-ko	+	-	+	-	-	+	+
	CD25-KO	-	+	-	+	+	-	-
	Nfkbiz	-	+	-	+	+	-	-
	IxBɑ	-	-	+	+	+	+	+
	RBAp48 Tg	+	+	+	+	-	+	+
	BAFF Tg	+	-	+	+	+	-	-
	IL-12 Tg	+	-	+	+	-	-	+
	IL-14a Tg	-	-	+	-	+	+	+
	IL-10 Tg	+	+	+	+	-	-	-
	Opn Tg	+	-	+	+	-	+	-
Induced mouse models	Salivary gland protein	+	-	+	-	-	-	-
	M3R	+	-	+	-	-	-	-
	Ro60 peptide	+	+	+	+	-	+	+
	Adenovirus 5	+	-	+	-	-	-	-
	Murine CMV	+	-	+	+	-	+	+
Humanized mouse model		+	-	+	+	-	-	-

‘-’ the characteristic is either absent or it has not been studied in this mouse model; ‘+’ the characteristic is present in this mouse model.

## Data Availability

Publicly available datasets were analyzed in this study and referred to in the list of references.
